# US Pediatric Primary Care Physician Workforce in Rural Areas, 2010 to 2020

**DOI:** 10.1001/jamanetworkopen.2023.33467

**Published:** 2023-09-13

**Authors:** Tarun Ramesh, Hao Yu

**Affiliations:** 1 Harvard Medical School, Boston, Massachusetts; 2 Department of Population Medicine, Harvard Medical School and Harvard Pilgrim Health Care Institute, Boston, Massachusetts

## Abstract

This cross-sectional study examines the growth and distribution of the general pediatrician and family medicine physician workforce from 2010 to 2020 across the US and identifies the sociodemographic characteristics of counties lacking this workforce.

## Introduction

Physician shortages are associated with substantial health disparities for pediatric populations in rural areas.^1^ Concerns about pediatric workforce shortages continue as pediatric residency applications from newly minted physicians decrease.^2^ In response, policymakers have taken considerable efforts to increase the pediatric workforce by funding the Children’s Hospital Graduate Medical Education program and through multiple provisions of the Patient Protection and Affordable Care Act, including the Community Health Fund, Health Center Appropriations, and School-Based Health Centers.^3,4^ However, it remains unknown whether the pediatric primary care physician workforce has grown substantially during the past decade. To fill the gap, we assessed the growth and distribution of the general pediatrician and family medicine physician (FMP) workforce from 2010 to 2020 across US counties. We then identified sociodemographic characteristics of counties lacking the workforce.

## Methods

This analysis used repeated cross-sectional data from 2010 to 2020. The data were publicly available and were determined as not human participants research by Harvard Pilgrim Health Care Institute; thus, informed consent was not needed, in accordance with 45 CFR §46. This study followed Strengthening the Reporting of Observational Studies in Epidemiology (STROBE) reporting guidelines for observational studies.

We used annual, county-level data about general pediatricians and FMPs from the Health Resources and Services Administration (HRSA) Area Health Resources Files and county characteristics from the American Community Survey 5-year estimates. We first described growth and distribution of general pediatricians and FMPs before performing a multivariable logistic regression on whether a county lacked both physician types in a year. The regression included state and year fixed effects, controlling for the following county-year level covariates: health insurance, kindergarten through 12th grade school enrollment, child poverty status, racial and ethnic composition, rurality (defined as 2013 US Department of Agriculture rural-urban continuum codes of 4, 5, 6, 7, 8, or 9), US Census region, and health professional shortage area (HPSA) designation by HRSA. All tests were 2-sided, used an α level of .05, and were conducted in Stata statistical software version 17.0 (StataCorp).

## Results

From 2010 to 2020, the number of general pediatricians increased by 6.0% (53 600 vs 56 800 pediatricians), but fewer of them worked in rural counties (3000 vs 2900 pediatricians). Similarly, the number of FMPs increased by 14.1% (79 400 vs 90 600 FMPs) with fewer working in rural counties (13 000 vs 12 000 FMPs). Despite a small increase in general pediatrician density (6.44 vs 6.96 pediatricians per 10 000 children), the density decreased marginally in completely rural counties (0.92 vs 0.81 pediatricians per 10 000 children). There were 2.8% more rural counties without a general pediatrician in 2020 vs 2010 (1156 vs 1124 counties) and 8.4% more rural counties without both general pediatricians and FMP (284 vs 262 counties) (Table 1).

**Table 1.** Changes in Pediatric Primary Care Workforce From 2010 to 2020^a^

Variable	2010	2020	Change, absolute (%)
General pediatricians, No.			
Total	53 600	56 800	3200 (6.0)
Working in rural counties	3000	2900	−100 (−3.3)
Working in completely rural counties	107	86	−21 (−19.6)
Family medicine physicians, No.			
Total	79 400	90 600	11 200 (14.1)
Working in rural counties	13 000	12 000	−1000 (−7.7)
Working in completely rural counties	1090	1057	−33 (−3.0)
General pediatrician density, No./10 000 children			
Total	6.44	6.96	0.52 (8.10)
Rural counties	2.48	2.59	0.11 (4.40)
Completely rural counties	0.92	0.81	−0.11 (−11.90)
Total counties without general pediatricians, No.	1386	1391	5 (0.4)
Counties without general pediatricians, %	44.1	44.3	0.2 (0.5)
Rural counties without general pediatricians, No.	1124	1156	32 (2.8)
Rural counties without general pediatricians, %	57.0	58.7	1.7 (3.0)
Completely rural counties without general pediatricians, No.	564	577	13 (2.3)
Completely rural counties without general pediatricians, %	88.3	90.4	2.1 (2.4)
Total counties without both general pediatricians and family medicine physicians, No.	304	331	27 (8.9)
Counties without both general pediatricians and family medicine physicians, %	9.7	10.5	0.8 (8.2)
Rural counties without both general pediatricians and family medicine physicians, No.	262	284	22 (8.4)
Rural counties without general pediatricians and family medicine physicians, %	13.3	14.4	1.1 (8.5)
Completely rural counties without both general pediatricians and family medicine physicians, No.	231	242	11 (4.8)
Completely rural counties without both general pediatricians and family medicine physicians, %	36.2	37.9	1.7 (4.7)
Total counties, No.	3141	3139	−2 (−0.1)
Total rural counties, No.	1971	1969	−2 (−0.1)
Total completely rural counties, No.	639	638	−1 (−0.2)

^a^ There has been an overall reduction in pediatricians working in rural areas. Data about physician distribution were derived from the Area Health Resources File in 2010 and 2020, while counties with US Department of Agriculture rural-urban continuum codes of 4, 5, 6, 7, 8, or 9 were included as rural counties. Completely rural counties were designated with US Department of Agriculture rural-urban continuum codes of 8 or 9.

Counties without both general pediatricians and FMPs were more likely to have higher percentage of non-Hispanic Black children (adjusted odds ratio [AOR], 1.01; 95% CI, 1.01-1.02), be rural (AOR, 1.45; 95% CI, 1.29-1.64), have higher child uninsured rates (AOR, 1.27; 95% CI 1.25-1.29), have higher child poverty levels (AOR, 1.02; 95% CI, 1.01-1.03), and have fewer children enrolled in kindergarten through 12th grade (AOR, 0.89; 95% CI 0.89-0.91). Notably, these counties were not associated with HPSA designation (Table 2).

**Table 2.** Characteristics of Counties Without Both General Pediatricians and Family Medicine Physicians^a^

Variable	Adjusted OR (95% CI)	*P* value
Race and ethnicity, percentage of children		
Hispanic	0.99 (0.99-1.00)	.02
Non-Hispanic, Black	1.01 (1.01-1.02)	<.001
Non-Hispanic, White	1 [Reference]	NA
Non-Hispanic, other^b^	1.00 (0.99-1.01)	.67
Percentage of children without health insurance	1.27 (1.25-1.29)	<.001
Percentage of children enrolled in kindergarten through 12th grade	0.89 (0.89-0.91)	<.001
Percentage of children living under Federal Poverty Level	1.02 (1.01-1.03)	<.001
Any health professional shortage area designation in a county	1.31 (0.83-2.05)	.24
Rurality		
Rural	1.45 (1.29-1.64)	<.001
Urban	1 [Reference]	NA
Census region		
Northeast	1 [Reference]	NA
Midwest	0.28 (0.08-1.02)	.05
South	0.46 (0.13-1.61)	.22
West	0.23 (0.06-0.96)	.04

Abbreviations: NA, not applicable; OR, odds ratio.

^a^ This state-year fixed effects, multivariable logistic regression shows that counties without general pediatricians or family medicine physicians were disproportionately Black, uninsured, and rural, with more children living under the Federal Poverty Level and fewer attending kindergarten through 12th grade at school. Data on physician distribution were extracted from Area Health Resources Files from 2010 to 2020, demographic and socioeconomic information were derived from the American Community Survey 5-year estimates at the county level, and data on rurality included US Department of Agriculture rural-urban continuum codes of 4, 5, 6, 7, 8, or 9.

^b^ Race and ethnicity were self-reported in the American Community Survey 5-year estimates. Non-Hispanic other refers to individuals who are Asian, Indigenous, multiracial, or marked *other* on the American Community Survey.

## Discussion

This cross-sectional study found that although numbers of general pediatricians and FMPs increased nationwide from 2010 to 2020, fewer practiced in rural counties, likely re-entrenching rural-urban disparities in pediatric outcomes.^5^ Coupled with the declining percentage of FMPs treating children, the pediatric primary care workforce deteriorated in rural areas.^6^

One possible explanation for the lack of an association between counties without both general pediatricians and FMPs and the HPSA designation was that the designation is for the general population, not specifically for the pediatric population. Policy makers should consider creating a designation for states to identify areas with general pediatrician and FMP shortages as pediatric HPSAs. A similar initiative, the Maternity Care Target Areas, was recently launched by HRSA to identify areas within HPSAs with few maternal care practitioners and deploy those types of clinicians accordingly. By targeting pediatric HPSAs, policymakers can strengthen the pediatric primary care physician workforce, especially in rural areas. Our study was limited by the lack of information about physicians in subcounty areas, which warrant further exploration.

## Notes

Supplement.
Data Sharing Statement
