# Supplementary material for: US Pediatric Primary Care Physician Workforce in Rural Areas, 2010 to 2020
Source: JAMA Netw Open. 2023 Sep 13;6(9):e2333467. doi: 10.1001/jamanetworkopen.2023.33467 (PMC10500373; doi:10.1001/jamanetworkopen.2023.33467)
Supplement: Supplement. — Data Sharing Statement [file jamanetwopen-e2333467-s001.pdf]

Data Sharing Statement

Ramesh. US Pediatric Primary Care Physician Workforce in Rural Areas, 2010 to 2020. *JAMA Netw Open*. Published September 13, 2023. doi:10.1001/jamanetworkopen.2023.33467

Data
Data available: Yes
Data types: Other (please specify)
Additional Information: This study used publicly available data, which can be downloaded at the websites below.
How to access data: Health Resources and Services Administration's (HRSA) Area Health Resources Files (AHRF) are available at: https://data.hrsa.gov/topics/health-workforce/ahrf
American Community Survey 5-year estimates are available at:
https://www.census.gov/data/developers/data-sets/acs-5year.html
When available: With publication

Supporting Documents
Document types: None

Additional Information
Who can access the data: anyone requesting the data
Types of analyses: For research only
Mechanisms of data availability: Without investigator support
